# Process Optimization and Odor Analysis of Instant Black Tea Powder

**DOI:** 10.3390/foods14091552

**Published:** 2025-04-28

**Authors:** Yuqin Xiong, Haomu Liao, Haiyue Liao, Xiaoyue Song, Chunhua Ma, Yan Huang

**Affiliations:** 1 College of Food Science, Fujian Agriculture and Forestry University, Fuzhou 350002, China; 52309010073@fafu.edu.cn (Y.X.); 52309010048@fafu.edu.cn (H.L.); sxysawyer@163.com (X.S.); 2 College of Tea and Food Sciences, Wuyi University, Wuyishan 354300, China; 19977413799@163.com

**Keywords:** instant black tea powder, process optimization, odor, electronic nose, gas chromatography–mass spectrometry

## Abstract

This study enhanced the odor retention of instant black tea powder by utilizing ultrasonic-assisted extraction and β-cyclodextrin embedding technology. Through single-factor tests considering variables such as the tea-to-water ratio, extraction temperature, ultrasonic extraction duration, and β-cyclodextrin addition, the optimal extraction conditions were determined. The ideal parameters were identified as follows: β-cyclodextrin was added at a rate of 7.5%, the tea-to-water ratio was 1:16, the ultrasonic extraction temperature was 52 °C, and the extraction duration was 30 min, and then the extract was processed by freeze-drying to obtain instant tea powder. Electronic nose trials revealed that the primary volatile odor compounds distinguishing the 14 groups of instant black tea soups were sulfides, terpenes, nitrogen oxides, alkanes, and aromatic compounds. HS-SPME-GC-MS analysis identified 65 effective volatile compounds, among which 11 key odor compounds, including Benzyl alcohol, Phytol, phenylethyl alcohol, 1,6,10-Dodecatrien-3-ol,3,7,11-trimethyl-,(E)-, Benzeneacetaldehyde, Undecanoic acid, ethyl ester, Dodecanoic acid, ethyl ester, Tetradecane, 2,4-Di-tert-butylphenol, 2-Pentadecanone, 6,10,14-trimethyl-, and indole, were the main contributors to the odor profile of instant black tea. The instant black tea powder produced under these conditions exhibited high quality, providing a valuable reference for further research on the production process of instant black tea powder.

## 1. Introduction

China, as the birthplace of black tea, boasts a rich and profound tea culture [1,2,3,4]. Black tea is one of the six basic types of tea in China and plays a pivotal role in the country’s tea production and export [5,6,7,8]. Summer and autumn teas have notably lower levels of amino acids and vitamins, while caffeine and polyphenol levels are much higher, which significantly diminish the balance of taste and odor compared to spring teas [9,10,11]. Despite the abundance of summer and autumn tea resources, their lower quality and underutilization lead to substantial resource waste [12].

Shuai Daliang et al. [13] used fresh summer and autumn tea leaves (one bud and two leaves) as raw materials to produce black tea, inoculating them with Fu brick tea bacterial liquid to create a unique red tea product combining the distinctive aromas of both black tea and goldenrod fungus. By fermenting black tea with the enzyme polyphenol oxidase found in apple, pear, or banana, Zou Chun et al. enhanced the quality and flavor of summer and autumn black tea, reducing its bitterness and astringency while imparting a sweet, fruity odor. Additionally, research has explored using summer and autumn tea to produce instant black tea concentrate, which is rich in nutrients, portable, and well suited for a fast-paced lifestyle [14,15]. The challenge remains to overcome the limitations of traditional instant tea processing techniques, minimize the loss of tea odor during production, preserve as much of the original black tea’s content and fragrance as possible, and improve the overall quality of instant tea [16,17].

The odor of tea soup is a critical factor influencing the quality of instant tea products [18,19,20,21,22,23]. β-cyclodextrin (β-CD) aids in the preservation and enhancement of odor by encapsulating the fragrant compounds in tea leaves, thereby reducing their loss during processing [4]. Yang Doudou et al. improved the solubility and rapid dissolution of mulberry leaf instant tea powder by incorporating 5% β-cyclodextrin as an embedding agent and extracting the leaves for 20 min [24]. In this study, ultrasonic-assisted extraction was used to shorten the extraction time. Instant black tea was prepared by the β-cyclodextrin embedding method with response surface optimization [25,26,27,28]. The quality of the prepared instant black tea powder was analyzed comprehensively by an electronic nose combined with HS-SPME-GC-MS [29,30,31], so as to provide a reference for further improving the quality of instant black tea [32,33].

## 2. Materials and Methods

### 2.1. Materials and Reagents

Black tea was obtained from Wuyishan Xuling Rock Tea Factory, Wuyishan City, Fujian Province, China; β-cyclodextrin (food grade) from Yunmo Biotechnology Co., Ltd., Shanghai City, China; Ethyl caprate (chromatography pure) from Shanghai Aladdin Biochemical Technology Co., Ltd., Shanghai City, China; and n-hexane (chromatography pure) from Shanghai Maclean’s Biochemical Technology Co., Ltd., Shanghai City, China.

### 2.2. Instruments and Equipment

The YF-6CHZ-2 Tea Roasting Machine was obtained from Fujian Anxi Yongfeng Machinery Co., Ltd., Anxi County, Quanzhou City, Fujian Province, China; FW80 high-speed grinder from Tianjin Tester Instrument Co., Ltd., Tianjin City, China; KQ5200DE CNC ultrasonic cleaner from Kunshan Ultrasonic Instrument Co., Ltd., Kunshan City, Jiangsu Province, China; RE-5205A rotary evaporator from Shanghai Yarong Biochemical Instrument Factory, Shanghai City, China; BUCHI L-200 Freeze Dryer from Shanghai Shujun Instrument Equipment Co., Ltd., Shanghai City, China; PEN3 electronic nose from Beijing Yingsheng Hengtai Technology Co., Ltd., Beijing City, China; DVB/CAR/PDMS solid-phase microextraction fibers from Shanghai Jingrui Scientific Instrument Effective Company, Shanghai City, China; and GCMS-TQ8050NX Gas Chromatography Mass Spectrometer from Shimadzu Instruments (Suzhou) Co., Ltd., Suzhou City, Jiangsu Province, China.

### 2.3. Test Method

#### 2.3.1. Instant Black Tea Powder Preparation Process

The production process of instant black tea powder consists of several key steps. Initially, black tea powder was prepared, followed by extraction. The extract was then filtered to remove impurities. Subsequently, rotary evaporation and concentration were performed to concentrate the tea extract. The concentrated extract was frozen at −80 °C and then subjected to vacuum freeze-drying to obtain the instant black tea powder. Finally, the product was stored in a sealed container to maintain its quality.

The specific process for producing instant black tea powder is as follows: First, remove the thick stems from the black tea, dry it at 80 °C for 30 min, and then grind it using a high-speed grinder and sieve it through a 40-mesh sieve. Accurately weigh 10 g of black tea powder (with an accuracy of ±0.001 g). Transfer 200 mL of purified water at 55 °C (solid-to-liquid ratio: 1:20) into an 800 mL beaker. Add β-cyclodextrin to the beaker, stir thoroughly, cover the beaker with plastic wrap, and proceed with ultrasonic extraction using an ultrasonic device set to 28 kHz at a constant temperature of 55 °C for 30 min. Simultaneously, perform a control experiment by conducting a static extraction under the same temperature conditions without adding β-cyclodextrin. Once the extraction is complete, filter and separate the tea powder and tea infusion using a 150-mesh nylon gauze. Then, transfer the filtered extract to an evaporating flask and concentrate it under vacuum for approximately 15 to 20 min until the solution becomes viscous. Place the concentrate into a 50 mL storage tube and freeze it at −80 °C for 18 h. To produce instant black tea powder, seal the frozen concentrate in an aluminum foil bag, puncture small holes in the bag, and freeze-dry it under low-temperature vacuum conditions for 96 h. Finally, seal the powder in a polyethylene bag and store it in a desiccator to maintain its quality.

#### 2.3.2. Extraction Single-Factor Optimization Test

Single-factor experiments were conducted to optimize the extraction process, taking into consideration the tea-to-water ratio (1:10, 1:15, 1:20, 1:25, 1:30), ultrasonic extraction time (10, 20, 30, 40, 50 min), ultrasonic extraction temperature (35, 45, 55, 60, 65 °C), and β-cyclodextrin addition levels (4%, 6%, 8%, 10%, 12%). Sensory evaluation scores were used as the assessment criteria, with each experiment repeated three times.

#### 2.3.3. Sensory Evaluation Method for Instant Black Tea

For each group, 1 g of instant black tea powder was brewed in 200 mL of purified water at 80 °C to prepare samples for testing. A sensory evaluation panel of 10 tea tasters followed the method described by Lai [34] and the standards in Q/QRYZ 0004 S-2022. The sensory evaluation standards are shown in Table 1.

#### 2.3.4. Design of Process Optimization Experiment Using Response Surface Methodology

Based on the results of single-factor experiments, response surface methodology (RSM) was employed to optimize the extraction process. The factors selected for optimization included the tea-to-water ratio (A), ultrasonic extraction temperature (B), and the amount of β-cyclodextrin added (C), with the sensory score as the dependent variable. The levels of these factors are shown in Table 2.

#### 2.3.5. Measurement of Instant Black Tea Odor Components Using Electronic Nose

Accurately weigh 0.2 g of instant black tea powder (±0.001 g) into a 50 mL headspace vial. Add 10 mL boiling purified water, seal the vial, shake gently, and let it stand for 30 min. Set the following electronic nose parameters: gas flow rate to 0.4 L/min, sensor cleaning time to 200 s, sample preparation time to 5 s, and sample collection time to 90 s. Set the operating environment’s temperature to room temperature. Perform three parallel measurements for each sample.

#### 2.3.6. Analysis of Odor Components and Content in Instant Black Tea Using HS-SPME-GC-MS

Accurately weigh 0.1 g of instant black tea powder (±0.001 g) into a 20 mL headspace vial. Add 5 mL of boiling purified water and 10 μL of a 50 μg/mL ethyl decanoate solution as the internal standard. Gently shake to mix, add a magnetic stir bar, seal the vial, and place it on a magnetic stirrer at 250 rpm and 75 °C for 10 min to equilibrate. Insert the extraction fiber into the headspace vial and extract for 50 min. After extraction, quickly remove the fiber and insert it into the gas chromatograph injection port. Desorb the sample at 250 °C for 3 min, and then perform GC-MS analysis [35].

The gas chromatography (GC) conditions are as follows: An HP-5MS capillary column (30 m × 250 µm × 0.25 µm) with a splitless injection mode, an injection port temperature of 250 °C, and high-purity helium gas (purity > 99.999%) as the carrier gas at a flow rate of 1.0 mL/min. A constant flow column flow control mode is used with a solvent delay of 3 min. The GC oven temperature program is as follows: hold at 50 °C for 1 min; increase to 80 °C at 2 °C/min and hold for 1 min; increase to 120 °C at 5 °C/min and hold for 1 min; increase to 140 °C at 4 °C/min and hold for 3 min; increase to 150 °C at 4 °C/min and hold for 3 min; increase to 160 °C at 5 °C/min and hold for 2 min; and finally, increase to 220 °C at 10 °C/min and hold for 5 min.

The mass spectrometry (MS) conditions are as follows: Electron impact ionization (EI) source with an electron energy of 70 eV, a transfer line temperature of 280 °C, an ion source temperature of 230 °C, and a quadrupole temperature of 150 °C. The scan mode is full scan (SCAN), with a mass scan range of *m*/*z* 45 to 600 amu.

The qualitative and semi-quantitative analysis of volatile odor compounds was performed using the NIST11 spectral library for compound identification, and the internal standard method was used for semi-quantification. Ethyl decanoate was used as the internal standard, and the concentration of volatile odor compounds was calculated using Equation (1):

(1) $$C_{x}=(S_{x}*C_{i})/S_{i}$$

where C_x_ and S_x_ represent the concentration and peak area of the volatile odor compound, respectively, and C_i_ and S_i_ represent the concentration and peak area of the internal standard ethyl decanoate, respectively.

### 2.4. Methods of Data Processing and Analysis

The response surface design and data analysis were performed using Design-Expert 13 software. The odor radar chart and odor component content chart were generated using Origin 2019 software. Additionally, principal component analysis (PCA) and loading analysis were conducted using Winmuster1.6.2 software.

## 3. Results and Discussion

### 3.1. Analysis of Different Processing Techniques for Instant Black Tea Powder

#### Single-Factor Test Results

**Figure 1:**
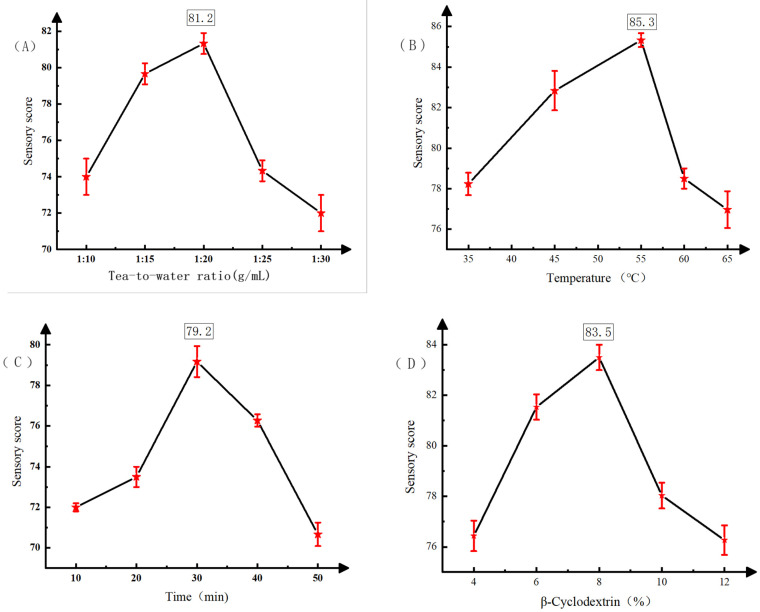
Single-factor experimental results. (**A**) Sensory rating of tea–water ratio; (**B**) sensory fractions of extraction temperature; (**C**) sensory fractions of extraction time; (**D**) sensory scores of β-cyclodextrin.

The single-factor results (Figure 1) show that the sensory score of instant black tea increased and then decreased with the tea-to-water ratio, peaking at 1:20. The score was highest at an ultrasonic extraction temperature of around 55 °C, while higher temperatures caused turbidity and odor loss. The optimal extraction time was 30 min. The tea’s color and odor retention were best when β-cyclodextrin was added at around 8%, achieving the highest sensory score.

### 3.2. Response Surface Test Results

#### 3.2.1. Design and Result Analysis of Response Surface Test

Based on the single-factor test results and in accordance with the Box–Behnken central composite design principle, a three-factor, three-level response surface optimization test was conducted. The factors selected for optimization included the tea-to-water ratio (A), ultrasonic extraction temperature (B), and β-cyclodextrin addition (C). The optimization results are shown in Table 3.

The data in Table 3 were analyzed using the response surface design software Design-Expert 13, with the sensory evaluation score Y as the response value. A multiple regression simulation was conducted, resulting in the following regression equation:

Y = 86.66 − 0.25A − 1.40B + 0.40C − 1.38AB + 2.42AC + 1.38BC − 2.12A^2^ − 3.02B^2^ − 3.27C^2^ (2)

The results of the regression model analysis are shown in Table 4. The regression model was highly significant (*p* < 0.0001) and the lack of fit was not significant (*p* = 0.1535 > 0.05), with a goodness of fit R^2^ = 0.9879 and the difference between R^2^Adj and R^2^Pre being less than 0.2, indicating that the model had good fit. The differences between the treatment conditions were significant, demonstrating that the three-factor, three-level analysis modeled by the equation is feasible, and the model is effective.

Based on the *p*-values, B (extraction temperature), interaction terms AB, AC, and BC, and quadratic terms A^2^, B^2^, and C^2^ all showed highly significant effects (*p* < 0.01) on the sensory score of instant black tea powder. Judging by the F-values, in the response surface optimization test, factor B (extraction temperature) exerts the most prominent influence on the quality of instant black tea powder.

#### 3.2.2. Interaction Analysis

**Figure 2:**
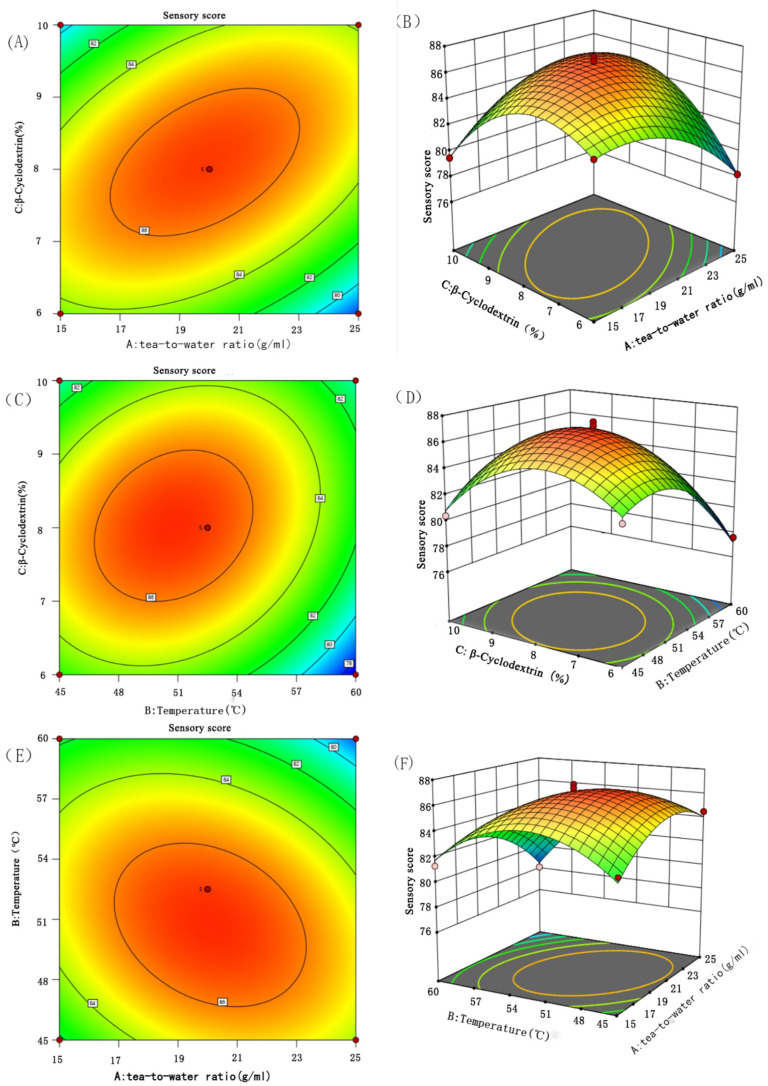
Response surface plots of the interaction effects of various factors on the sensory score of instant tea. (**A**) Response surface plane of tea–water ratio and β-cyclodextrin content to sensory scores of instant tea; (**B**) three-dimensional response surface of tea–water ratio and β-cyclodextrin content to sensory scores of instant tea; (**C**) response plane of extraction temperature and β-cyclodextrin content to sensory scores of instant tea; (**D**) three-dimensional response surface of extraction temperature and β-cyclodextrin content to sensory scores of instant tea; (**E**) response surface plane of extraction temperature and tea–water ratio to instant tea sensory score; (**F**) three-dimensional response surface of extraction temperature and tea–water ratio to sensory score of instant tea.

The response surface graph is a curved surface graph in three-dimensional space formed by the response values and various experimental factors. In the response surface analysis graph, the optimal parameters and the interactions between various parameters can be vividly observed. In Figure 2, a graphical analysis of the influence of the interactions among the three factors (A: tea-to-water ratio; B: ultrasonic extraction temperature; C: β-cyclodextrin) on the sensory score of instant black tea can be seen. It can be observed that in the interaction of A and C, the extreme value is very obvious, and the impact on the sensory score is particularly significant. As A and C increase, the sensory score of instant black tea powder first increases and then decreases. In a comparison of these three sets of response surface graphs, it can be clearly seen that the interaction between A and C has the most significant impact on the sensory score of instant black tea powder, followed by the interactions between B and C and between A and B. This is consistent with the results of the variance analysis.

#### 3.2.3. Verification of the Response Surface Model

The optimal processing conditions for instant black tea powder were obtained using Design-Expert 13 software to optimize and predict the experimental data. The predicted optimal conditions were as follows: a tea-to-water ratio of 1:15.7, an ultrasonic extraction temperature of 51.7 °C, an ultrasonic extraction time of 30 min, and a β-cyclodextrin addition of 7.4%. Under these conditions, the predicted sensory score for the instant black tea powder was 87.55 points. Considering practical operational constraints, the conditions were slightly adjusted to a tea-to-water ratio of 1:16, an ultrasonic extraction temperature of 52 °C, an ultrasonic extraction time of 30 min, and a β-cyclodextrin addition of 7.5%. Under these adjusted conditions, the measured sensory score was 88.7 points, which closely aligns with the theoretical prediction.

### 3.3. Results of Analysis of Instant Black Tea with Different Processing Methods by Electronic Nose Processing Techniques

#### 3.3.1. Analysis of the Odor Source of Instant Black Tea by Electronic Nose Sensors

**Figure 3:**
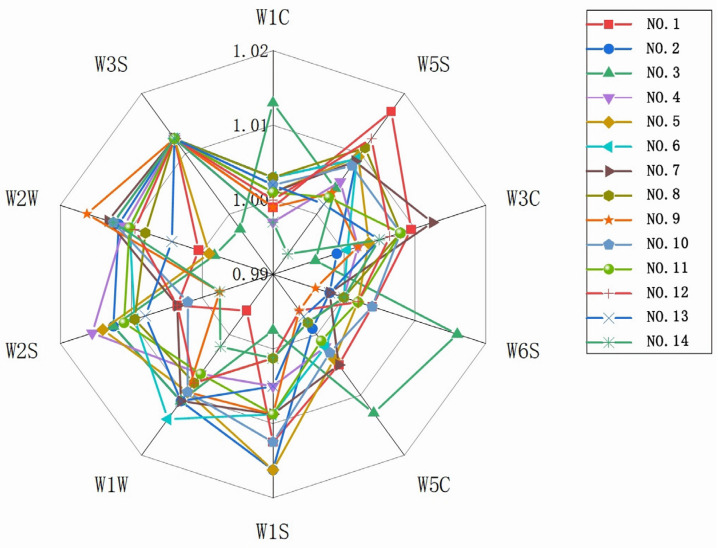
Radar chart of electronic nose sensor response values for instant black tea powder produced with different processing methods.

An electronic nose equipped with 10 different semiconductor metal oxide sensors was used to measure the odor of instant tea. The sensors included W1C, W5S, W3C, W6S, W5C, W1S, W1W, W2S, W2W, and W3S. The substances corresponding to the response of each sensor are listed in Table 5. During sample analysis, the main volatile substances in the sample could be identified based on the type of gas detected. Using response surface design, we analyzed 12 experimental points, 1 center point, and a blank control of instant black tea. The analysis examined the types and concentrations of odor compounds in 14 groups of instant tea processed using different methods, as shown in Figure 3.

As shown in Figure 3, Group 1 exhibits a higher content of nitrogen oxides, as indicated by its prominent response in W5S. Group 3 shows elevated values in W1C, W6S, and W5C, suggesting a higher content of aliphatic aromatic compounds, benzene series, hydrogenated compounds, short-chain alkanes, and other aromatic substances. Group 6 stands out in W1W, indicating a higher content of sulfides and terpenes. Group 9 is prominent in W2W, suggesting a higher content of organic sulfides and aromatic compounds. Additionally, most samples show relatively low and concentrated response values in the W6S, W5C, and W1C sensors, with some values close to 1, indicating minimal differences among these samples. In contrast, the W3C sensor detects substances with response values mostly greater than 1, except for Groups 2 and 3, which have relatively lower values below 1. The response values for different samples are highly concentrated in the W3S sensor, indicating that the long-chain alkane volatile substances in instant black tea produced by different processing methods do not vary significantly. In the W5S, W1S, W2W, W2S, and W1W sensors, most response values are greater than 1, suggesting that the prominent odor substances in instant black tea powder are primarily alkane aromatic substances, aromatic volatile substances, aldehydes, alcohols, and ester volatile substances. The blank control group (Group 14) shows significantly lower response values for volatile odor substances compared to the other 13 groups, indicating that ultrasonic-assisted extraction and the addition of β-cyclodextrin can effectively retain volatile odor substances during processing.

#### 3.3.2. Principal Component Analysis of the Odor of Instant Black Tea Soup

**Figure 4:**
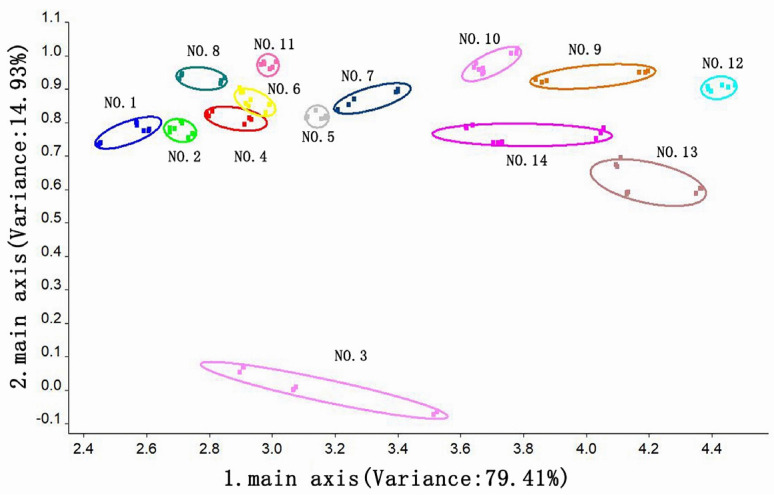
PCA plot of instant black tea soup.

Principal component analysis (PCA) was performed on fourteen groups of instant black tea soup processed using different methods, and the results of the odor substance analysis are displayed in Figure 4. PCA can effectively capture the distinctive characteristics of a sample when the total contribution rate exceeds 85%. In this case, the first and second principal components accounted for 79.41% and 14.93% of the variance, respectively, contributing to a total of 94.34%, which exceeds 85% (Figure 4). The odor substances in the 14 groups of instant black tea soup showed a clear separation trend along the first principal component. The most prominent odor substances were found in Groups 1 and 12, with nitrogen oxides and long-chain alkanes being the dominant compounds, respectively. From an elliptical distance perspective, Groups 4 and 6 exhibited a close, cross-correlated distance, indicating that their odor characteristics might be similar. The analysis of the second principal component revealed significant differences between Group 3 and the other groups in terms of odor compound content, suggesting that the processing method used for Group 3 altered the type and content of odor compounds in the instant black tea. Overall, the differences in odor components across the instant black tea powder samples were primarily influenced by variations in the β-cyclodextrin addition amount, ultrasonic extraction temperature, and tea-to-water ratio during processing.

#### 3.3.3. Loading Analysis of the Odor of Instant Black Tea Soup

**Figure 5:**
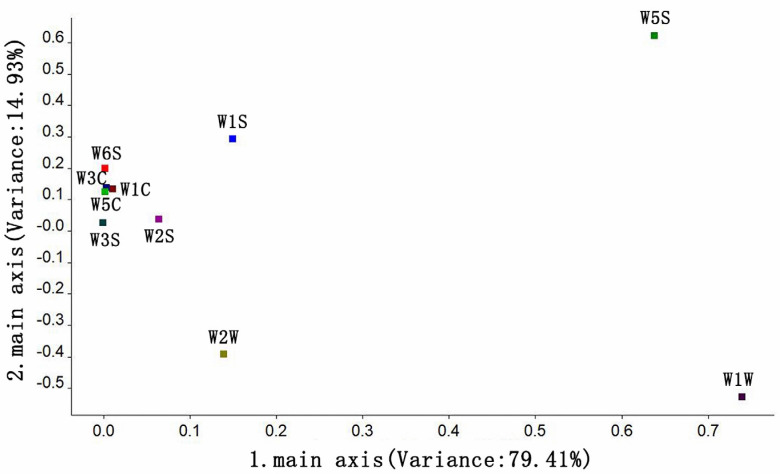
Loading analysis of instant black tea soup.

To determine which types of odor substances play a primary role in distinguishing the samples, a loading analysis of the electronic nose was conducted to reflect the contribution of various sensors in identifying odor substances in instant black tea and its soup. The analysis results are shown in Figure 5. Sensors closer to the origin have a smaller impact on the odor substances, while those farther from the origin have a greater effect.

The first and second principal components of the loading analysis accounted for 94.34% of the total variance (Figure 5). The sensors W1W and W5S made the greatest contribution to the first principal component, indicating that the dominant volatile substances in the odor of instant tea soup were alkanes, methane, methyl compounds, and nitrogen oxides. The sensors W5S, W2W, and W1S contributed more to the second principal component, suggesting that the odor components contained higher amounts of nitrogen oxides, alcohols, aldehydes, and ketones, as well as alkanes, methane, and methyl groups. The loading factors of the W6S, W3C, W1C, W5C, W2S, and W3S sensors were close to the origin, indicating that these sensors had low sensitivity to the volatile compounds in the odor of instant tea soup. The analysis revealed that sulfides, terpenes, nitrogen oxides, alkanes, and aromatic compounds were the primary volatile odor compounds distinguishing the 14 groups of instant black tea soup.

### 3.4. HS-SPME-GC-MS Detection and Analysis of the Volatile Components and Contents of Instant Black Tea

#### Analysis of Volatile Compounds Characteristics of Different Instant Teas

Through the use of headspace solid-phase microextraction–gas chromatography–mass spectrometry (HS-SPME-GC-MS), a total of 65 volatile compounds were detected, including 17 aldehydes, 8 alcohols, 15 esters, 12 alkanes, 4 ketones, 4 acids, 2 phenols, and 3 other compounds. The analysis showed that the primary volatile compounds of instant black tea produced by various methods are aldehydes, followed by esters, alkanes, and alcohols. Instant black tea retained the same sweet, floral, and fruity aromas as original black tea. Aldehydes typically exhibit floral notes, alcohols have unique floral and fruity aromas, esters contribute a fruity scent, and ketones provide a floral, sweet, and fruity odor, and these odor types are present in higher concentrations in each group of instant tea [36,37,38,39,40].

**Figure 6:**
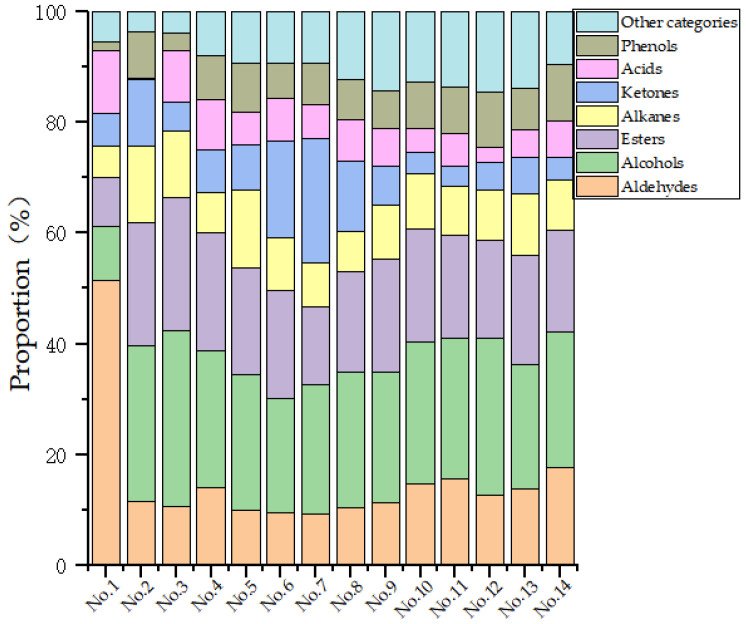
Relative content of volatile compounds in 14 groups of instant black tea.

As shown in Table 6, HS-SPME-GC-MS detected 65 volatile compounds, with 11 key odor substances—Benzyl alcohol, Phytol, Phenylethyl alcohol 1,6,10-Dodecatrien-3-ol,3,7,11-trimethyl-,(E)-, Benzeneacetaldehyde, Undecanoic acid, ethyl ester, Dodecanoic acid, ethyl ester, Tetradecane, 2,4-Di-tert-butylphenol, 2-Pentadecanone, 6,10,14-trimethyl-, and indole—having concentrations generally above 500 μg/kg. These compounds play a crucial role in the formation of the odor of instant black tea. Among most of the volatile compounds in instant black tea, alcohol compounds had the highest relative concentration, followed by esters and alkanes (Figure 6), both of which significantly contribute to the tea’s odor, consistent with the loading analysis. In Group 7, ketone compounds showed a relatively higher concentration, while alkanes were lower in this group. Additionally, aldehydes were most prominent in Group 1, where alcohols were present in the lowest relative concentration. These findings demonstrate that the odor components vary depending on the processing conditions. The results suggest that using Wuyi Mountain summer and autumn tea leaves and incorporating β-cyclodextrin in the production of instant black tea powder help retain the odor of the tea soup.

## 4. Conclusions

This study explored the improvement of the instant black tea powder production process using summer and autumn tea combined with β-cyclodextrin embedding technology. Sensory evaluation and response surface analysis revealed that the optimal processing conditions for instant black tea powder were as follows: a tea-to-water ratio of 1:16, an ultrasonic extraction temperature of 52 °C, an extraction time of 30 min, and a β-cyclodextrin addition of 7.5%. Under these conditions, the sensory score reached 88.7 points. The content of volatile odor compounds, taste, tea color, solubility, odor, and appearance of the instant black tea powder were all superior to those of the control group.

The principal component analysis (PCA) and loading analysis of electronic nose detection effectively distinguished the volatile substances under different processing conditions. The odor radar chart response values indicated that the primary odor compounds in the instant black tea powder were alkanes, aromatic volatile substances, aldehydes, alcohols, and ester volatile compounds. Through the use of headspace solid-phase microextraction–gas chromatography–mass spectrometry (HS-SPME-GC-MS), 65 effective volatile volatile compounds were detected across 14 groups of instant black tea. These components included alcohols, esters, aldehydes, ketones, acids, and alkanes, with high levels of esters and alcohols contributing to the tea’s distinctive floral and fruity odor. The results indicated that the odor components and their concentrations varied under different processing conditions.

This study concludes that the use of summer and autumn tea combined with the β-cyclodextrin embedding process enhanced the odor types and intensity of instant black tea powder. This approach allows for the greater utilization and development of summer and autumn tea resources, thereby improving the overall quality of instant black tea powder. These findings provide valuable reference data for future research on the production process of instant black tea powder. Future research could further explore the impact of β-cyclodextrin on odor components.

## Figures and Tables

**Table 1:** Sensory evaluation table for instant black tea powder.

Evaluation Item	Scoring Criteria	Score Range
Odor (30 points)	Dull, impure odor Pure odor Sweet, floral, or fruity odor	1~10 11~20 21~30
Taste (30 points)	Bland, slightly astringent taste Fairly mellow, but lacking smoothness Mellow, sweet, refreshing, with lingering aftertaste	1~10 11~20 21~30
Liquid Color (20 points)	Dark red, slightly cloudy Red, fairly bright Brownish red or reddish brown, clear and bright	1~6 7~13 14~20
Solubility (10 points)	Needs stirring to dissolve, sediment at the bottom Instantly dissolves, no sediment	1~5 6~10
Appearance (10 points)	Solid granules, dull, not easily dispersible Loose flakes or powder, glossy, no clumps or impurities	1~5 6~10

**Table 2:** Factors and levels of response surface experiments.

Level	Factor		
	A Tea-To-Water Ratio	B Temperature	C β-Cyclodextrin (%)
−1	1:15	45	6
0	1:20	55	8
1	1:25	60	10

**Table 3:** Response surface design scheme and test results for optimizing the processing of instant black tea powder.

Test Group	A	B	C	Sensory Score Y (Points)
1	0	−1	−1	82.3
2	0	−1	1	80.4
3	−1	−1	0	82.2
4	1	−1	0	84.5
5	−1	0	−1	83.6
6	1	0	−1	78.2
7	0	0	0	86.4
8	−1	0	1	79.5
9	1	0	1	83.8
10	0	1	−1	77.6
11	−1	1	0	81.3
12	1	1	0	78.1
13	0	1	1	81.2
14	0	0	0	86.2
15	0	0	0	87.2
16	0	0	0	86.9
17	0	0	0	86.6

**Table 4:** Regression model variance analysis results.

Source of Variance	Sum of Squares	Degree of Freedom	Mean Square	F-Value	*p*-Value	Significance
Model	169.78	9	18.86	63.27	<0.0001	**
A—tea–water ratio	0.5000	1	0.5000	1.68	0.2364	
B—temperature	15.68	1	15.68	52.59	0.0002	**
C—β-cyclodextrin	1.28	1	1.28	4.29	0.0770	
AB	7.56	1	7.56	25.37	0.0015	**
AC	23.52	1	23.52	78.90	<0.0001	**
BC	7.56	1	7.56	25.37	0.0015	**
A2	18.88	1	18.88	63.32	<0.0001	**
B2	38.34	1	38.34	128.59	<0.0001	**
C2	44.95	1	44.95	150.78	<0.0001	**
Residuals	2.09	7	0.2981			
Lack of fit	1.45	3	0.4850	3.07	0.1535	Not significant
Pure error	0.6320	4	0.1580			
Total	171.87	16				
R^2^	0.9879					
Adjusted R^2^	0.9722					
Predicted R^2^	0.8588					
Adeq precision	22.5893					

Note: *p* < 0.05 is a significant difference, marked with *; *p* < 0.01 is the most significant difference, and the mark is **.

**Table 5:** Substances measured by sensors.

Sensor	Determination of the Substance
W1C	Aliphatic aromatic compounds, benzene series
W5S	Nitrogen oxides
W3C	Amines, aromatic substances
W6S	Hydrogenated compounds
W5C	Short-chain alkanes, aromatic substances
W1S	Alkanes, methane, methyl compounds
W1W	Sulfides, terpenes
W2S	Alcohols, aldehydes, ketones, aromatic compounds
W2W	Organic sulfides, aromatic substances
W3S	Long-chain alkanes

**Table 6:** Composition and content of volatile compounds substances in 14 groups of instant black tea powder (μg/kg).

Number	Compounds	No.1	No.2	No.3	No.4	No.5	No.6	No.7	No.8	No.9	No.10	No.11	No.12	No.13	No.14
	Aldehydes														
1	Benzaldehyde	210.0	319.3	254.4	370.7	440.7	310.6	138.4	541.7	569.2	659.9	885.7	646.9	289.6	591.0
2	(E,E)-2,4-Heptadi-enal	161.0	566.3	356.9	366.2	94.4	300.3	288.0	400.3	293.8	614.0	522.0	524.8	260.9	354.8
3	Benzeneacetaldehyde	165.7	926.7	815.5	1266.9	870.5	976.5	1065.1	1021.8	1310.0	1128.5	1307.7	1327.0	994.2	1586.7
4	2-Undecenal	368.7	404.5	215.6	500.0	122.7	168.4	439.7	570.7	395.4	156.0	362.6	174.4	562.9	436.9
5	Dodecanal	16,462.8	437.5	346.4	540.7	403.0	419.6	413.7	387.4	474.0	517.8	513.7	438.1	359.4	474.2
6	2,4-Nonadienal, (E,E)-	108.9	108.9	56.7	109.6	57.3	92.9	122.7	132.2	46.8	865.1	97.2	71.9	28.8	34.7
7	Tetradecanal	20,155.6	129.3	292.1	185.3	100.2	199.1	100.6	67.3	168.1	210.8	106.2	164.3	-	270.8
8	Nonanal	169.7	166.8	119.7	269.3	152.5	89.2	208.0	236.3	347.5	151.0	235.4	198.5	172.1	183.5
9	Undecanal	8291.3	66.2	48.1	84.5	51.5	26.3	71.2	64.9	89.4	65.9	74.0	77.8	65.3	91.2
10	Decanal	335.3	188.2	126.9	287.4	154.0	167.3	225.1	202.6	257.3	160.2	212.4	197.8	147.5	203.6
11	2-Hexenal	43.8	51.2	31.1	74.3	-	-	46.7	51.7	50.9	35.2	46.1	-	-	182.0
12	2-Octenal, (E)-	20.5	24.4	13.3	63.6	12.7	30.5	14.6	49.5	17.7	14.1	27.8	-	-	-
13	2-Nonenal, (E)-	30.7	34.4	39.5	83.1	43.5	51.7	79.1	85.4	89.2	51.6	78.1	59.4	26.1	66.8
14	Tridecanal	79.3	98.3	76.9	97.4	192.1	139.1	182.6	175.9	85.0	152.5	153.9	95.4	147.5	183.4
15	(E)-Tetradec-2-enal	1372.1	-	-	-	-	-	-	-	-	9.3	-	-	-	-
16	13-Methyltetradecanal	500.0	91.6	31.2	-	-	-	-	122.1	95.6	-	104.7	-	213.9	-
17	(Z)-9-Hexadecenal	1722.1	-	-	-	-	-	-	-	-	-	-	-	-	-
	Alcohols														
18	Benzyl alcohol	578.1	566.3	491.2	842.3	264.3	553.0	777.5	465.1	436.0	435.9	871.8	795.6	605.0	1200.2
19	Phenylethyl alcohol	1476.7	1830.4	1543.9	2596.6	1793.1	2080.1	2529.9	2293.9	2749.9	2295.8	2587.0	2489.0	1990.2	3273.9
20	Phytol	2183.1	3038.8	3187.5	4075.1	4536.6	4097.1	4138.2	6252.1	4541.8	4339.0	4462.2	4039.7	4173.9	2710.9
21	2,6,10-Dodecatrien-1-ol, 3,7,11-trimethyl-	266.7	511.5	424.1	274.4	505.1	235.0	562.9	599.8	490.3	455.0	583.8	538.4	430.0	505.6
22	Geraniol	121.1	750.2	195.4	319.2	184.9	279.1	812.7	400.8	648.4	661.8	760.7	827.5	375.3	697.4
23	1,6,10-Dodecatrien-3-ol, 3,7,11-trimethyl-,(E)-	2554.4	4941.5	4818.1	3931.5	4086.0	3507.0	5721.1	5611.4	4306.9	4277.0	4885.6	7281.7	3803.5	4641.2
24	Linalool	20.9	232.6	55.2	38.6	39.5	26.7	95.2	38.5	76.4	210.5	253.3	268.6	31.4	132.5
25	2-Decenal, (E)-	-	424.2	118.3	193.6	128.5	251.5	348.2	395.3	280.7	135.2	190.6	170.6	198.6	212.4
	Esters														
26	Propyl decanoate	2177.5	544.2	713.6	634.9	718.0	489.9	-	-	-	522.4	479.6	506.3	829.3	504.2
27	Undecanoic acid, ethyl ester	1872.9	2846.7	2634.8	3697.6	2713.5	2776.2	2390.2	2848.5	2816.9	3577.6	3323.6	3736.8	3006.4	3257.6
28	Dodecanoic acid, ethyl ester	875.7	1398.6	1276.3	1610.3	1369.2	1308.4	1233.4	1440.2	1409.2	1743.4	1543.4	1702.5	1433.3	1399.1
29	9,12-Octadecadienoic acid (Z,Z)-, methyl ester	64.02	44.6	-	42.67	130.54	19.48	74.82	51.71	67.43	59.58	35.42	94.52	28.0	56.0
30	Hexadecanoic acid, methyl ester	1986.4	356.7	358.4	409.5	398.2	622.3	493.2	498.8	383.4	425.6	418.6	428.9	461.1	311.3
31	1,4-Dibutyl benzene-1,4-dicarboxylate	-	-	270.8	452.7	414.6	534.9	526.4	561.6	2714.1	326.9	427.8	425.3	340.6	422.4
32	Isopropyl palmitate	1111.3	115.7	-	186.3	41.8	135.4	-	-	290.0	75.4	69.0	-	100.1	33.2
33	1,2-Benzenedicarboxylic acid, bis(2-methylpropyl) ester	199.04	469.79	310.8	-	453.91	547.68	524.08	538.17	335.11	194.9	188.11	267.17	193.24	386.47
34	Pentanedioic acid, dimethyl ester	98.0	127.9	148.0	70.8	98.2	100.4	131.7	271.7	205.6	199.3	267.5	75.8	140.4	246.5
35	Bis(2-ethylhexyl) phthalate	19.92	37.43	-	-	26.27	55.2	88.89	114.01	64.89	33.74	23.5	18.27	35.62	71.23
36	Hexanedioic acid, dimethyl ester	136.6	207.7	83.0	108.5	194.9	180.1	243.8	369.2	260.9	241.0	286.7	103.7	184.0	304.0
37	.gamma.-Tetradecalactone	4593.1	-	-	-	-	924.2	-	1749.3	439.0	-	-	-	-	-
38	.beta.-Phenylethyl butyrate	-	245.7	42.0	87.0	-	370.4	-	-	-	-	-	-	-	-
39	Nerolidyl acetate	961.3	-	-	-	-	-	-	-	-	5165.7	4420.5	-	-	-
	Alkanes														
40	Tetradecane	1379.8	1157.9	1532.0	1091.8	2027.0	1191.5	1123.9	1103.1	1112.6	1034.3	991.3	1171.4	1078.6	1067.9
41	Pentadecane	815.7	717.2	554.0	852.6	711.7	961.8	730.9	842.2	1172.1	992.4	847.5	988.8	878.6	1049.2
42	Eicosane	184.7	287.9	243.9	392.0	305.6	454.4	346.1	413.5	432.2	363.3	409.9	395.0	328.3	447.2
43	Heptadecane	146.84	93.44	-	96.55	206.41	111.93	95.19	116.87	89.2	98.23	95.19	196.87	229.27	185.43
44	Nonadecane	103.51	103.51	-	93.99	315.32	216.4	280.11	172.86	246.28	213.88	196.96	216.4	213.88	196.96
45	Heptadecane, 3-methyl-	-	-	-	-	188.27	75.51	139.22	63.27	61.47	139.22	75.51	139.22	75.51	-
46	Hexadecane	-	-	-	96.39	83.91	-	-	-	-	-	-	-	-	-
47	Pentadecane, 2,6,10,14-tetramethyl-	-	-	-	-	-	101.27	107.47	140.61	135.47	144.26	102.33	101.27	101.27	-
48	2-Methyltetracosane	427.3	141.2	132.6	120.3	109.8	185.1	155.8	116.6	173.8	144.8	184.4	187.0	190.6	196.3
49	11-Methyltricosane	804.3	-	-	-	-	-	-	23.6	112.3	107.2	114.0	219.4	97.6	98.1
50	2,6,10-Trimethyltridecane	-	-	-	100.55	108.11	100.07	108.65	100.07	108.65	108.65	108.65	108.65	81.71	-
51	2-Methylhexacosane	404.8	141.2	53.7	66.7	109.8	267.5	155.8	251.8	197.4	144.8	187.8	136.6	190.6	-
52	.beta.-Phenylethyl butyrate	-	245.7	42.0	87.0	-	370.4	-	-	-	-	-	-	-	-
	Ketones														
53	γ-Dodecalactone	267.5	-	-	650.0	-	577.6	-	717.4	663.4	530.0	339.5	318.3	327.6	308.6
54	2-Pentadecanone, 6,10,14-trimethyl-	2885.1	1533.7	444.1	1389.2	1051.6	3595.6	4870.5	2462.2	1017.7	490.0	520.5	893.4	879.9	597.2
55	.alpha.-Ionone	110.9	125.7	125.4	154.8	-	113.0	-	-	201.2	304.8	315.9	316.9	-	340.6
56	2(4H)-Benzofuranone, 5,6,7,7a-tetrahydro-4,4,7a-trimethyl-, (R)-	-	201.6	103.8	-	460.8	238.5	609.0	578.6	509.2	472.0	489.2	518.4	345.8	568.3
	Acids														
57	*n*-Decanoic acid	-	-	1166.8	1754.6	1144.6	1451.8	1492.5	1781.1	1506.0	873.1	1298.5	616.6	863.1	1540.9
58	Pentadecanoic acid	-	-	-	-	-	237.24	-	-	-	-	-	181.97	-	-
59	Eicosanoic acid	6035.7	75.7	71.2	64.1	59.8	-	-	-	-	-	68.2	63.4	55.3	69.9
60	Nonanoic acid	72.0	52.1	-	63.4	-	51.9	-	-	151.6	-	47.8	40.4	105.7	59.8
	Phenols														
61	2,4-Di-tert-butylphenol	757.0	1250.3	303.9	1488.9	1466.6	1225.5	1561.0	1561.2	1496.8	1514.7	1570.9	1402.1	1285.4	2167.8
62	Phenol, 2,4,6-tri-tert-butyl-	88.6	59.1	86.7	81.5	172.8	173.7	231.3	212.2	146.2	165.8	179.4	213.9	125.9	235.4
	Other categories														
63	Indole	1088.3	581.5	555.1	806.7	637.4	748.9	907.4	880.4	1086.6	791.4	985.8	873.5	691.0	957.3
64	Caffeine	701.8	-	512.4	613.8	993.5	1227.0	1385.1	1994.1	2442.4	865.4	1202.6	1562.0	988.1	1140.2
65	Neophytadiene	1664.1	1118.4	1386.4	1871.2	1868.7	1842.6	1786.2	2056.8	1725.1	1740.0	2020.0	1596.0	1468.3	1181.2

Note: “-” indicates that the compound has not been detected in this sample.

## Data Availability

The original contributions presented in the study are included in the article; further inquiries can be directed to the corresponding authors.
